# Cutaneous pseudolymphoma successfully treated with intralesional triamcinolone acetonide: A case report

**DOI:** 10.1177/2050313X241311362

**Published:** 2025-01-08

**Authors:** Nicole Asamoah, Lara Gunton

**Affiliations:** 1 Michael G. DeGroote School of Medicine, McMaster University, St. Catharines, ON, Canada; 2 Division of Dermatology, Department of Medicine, McMaster University, Hamilton, ON, Canada

**Keywords:** Cutaneous pseudolymphoma, treatment, triamcinolone acetonide

## Abstract

Cutaneous pseudolymphoma refers to a group of skin conditions that simulate lymphoma either clinically and/or histologically. Cutaneous pseudolymphoma is a benign disorder that can often be misdiagnosed and has a wide range of treatment modalities. Currently, there is no gold standard of treatment, and the literature would benefit from more reports on successful and unsuccessful treatments of cutaneous pseudolymphoma. In this case, we present a 24-year-old female with cutaneous pseudolymphoma successfully treated with intralesional triamcinolone acetonide after misdiagnosis and several failures with other treatments.

## Introduction

Cutaneous pseudolymphoma (CPL) refers to a group of skin conditions that simulate lymphoma either histologically and/or clinically through a benign lymphoproliferative reactive process. Most cases of CPL are idiopathic^1^; however, various causative agents can be broadly divided into four categories: infections (e.g., Borrelia species), drugs (e.g., nonsteroidal anti-inflammatory drugs), foreign agents (e.g., Tattoo ink), and others (e.g., UV radiation).^2^ Unlike cutaneous lymphomas that use the World Health Organization–European Organization for Research and Treatment of Cancer classification system, there is no consensus-based system to classify CPL. Nonetheless, the literature proposes different approaches to classifying CPL and notes that treatment is dependent on the subtype and/or the etiology. Consequently, there is no gold standard of treatment for CPL making it quite difficult to manage. Current known treatments produce variable responses in patients^1^ making it more challenging to manage. In this report, we present a case of CPL successfully treated with intralesional triamcinolone acetonide.

## Case report

**Figure 1.**
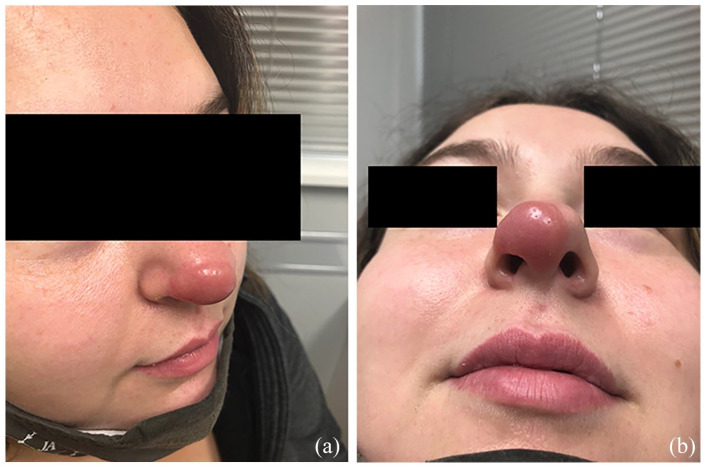
Prior to treatment with intralesional triamcinolone acetonide: (a) Lateral view, (b) Inferior view.

**Figure 2.**
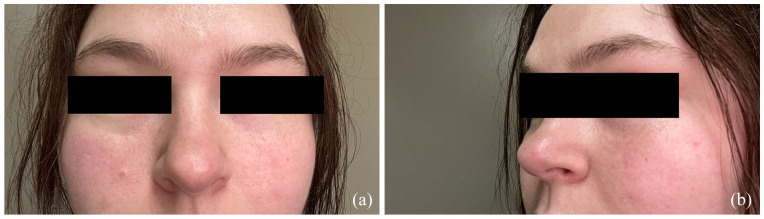
Two years after treatment with intralesional triamcinolone acetonide: (a) Anterior view (b) Lateral view.

A healthy 24-year-old woman presented to a walk-in clinic with a rapidly expanding non-tender pink lesion on the nasal tip. She was given a course of trimethoprim/sulfamethoxazole and fucidin and instructed to follow up in the emergency department if her symptoms worsen. Five weeks later, she presented to the emergency department with a painful, rapidly expanding well-demarcated flesh-colored plaque on her nose. The otolaryngology physician on call was consulted and recommended a trial of cephalexin and doxycycline. She was referred to dermatology and assessed 2 weeks later. On examination at the community dermatology office, a smooth, pink edematous firm plaque on the nasal tip extending down onto the columella was observed with no telangiectasias on dermoscopy (Figure 1). A specimen via punch biopsy was obtained and revealed a lymphohistiocytic infiltrate in the entire dermis and a slight spongiotic epidermis with exocytosis of atypical lymphocytes. The immunostains were consistent with T-cell lymphoid hyperplasia of unknown cause with most lesional cells staining positive for CD4 and CD8 and negative for CD30. Treatment via intralesional steroids was discussed and agreed upon by the patient, and 0.4 ml of 2.5 mg/ml of triamcinolone acetonide was administered to the lesion. At her 1-month follow-up, the lesion had improved significantly. The plaque had reduced a 50% in size and nearly all the redness had subsided. Repeat intralesional triamcinolone acetonide injection (0.3 ml of 2.5 mg/ml) was administered. Both injections were well tolerated with no side effects. One month after the repeat injection, the lesion was almost completely flat and did not require further treatment. Eight months after the first injection, the lesion had complete resolution and has remained stable over the last 2 years (Figure 2).

## Discussion

CPL is a condition that mimics cutaneous lymphoma either clinically or histologically. Diagnosis of the disease can be difficult and often misdiagnosed.^3^ In our case, the patient had been seen by three physicians prior to being worked up for suspected CPL. It is important for clinicians to have CPL as a differential diagnosis as the lesions can progress to malignant lymphoma.^4^ A diagnostic biopsy should be considered when diagnostic uncertainty occurs.

In addition, management of this condition is widespread with many different modalities being described in the literature ultimately creating challenges when treating CPL. The literature highlights successful treatment of CPL with rituximab,^3^ hydroxychloroquine,^5^ and dupilumab^6^ to name a few; however, there is no gold standard of treatment due to the various etiologies of the disorder. In this case, intralesional triamcinolone acetonide successfully treated CPL after failure with multiple other treatments.

Triamcinolone acetonide is an anti-inflammatory agent. It alters gene expression to induce anti-inflammatory proteins and inhibit inflammatory mediators.^7^ CPL is an inflammatory response to either specific antigens or unknown stimuli. Therefore, the response of CPL to intralesional steroid injection with triamcinolone acetonide may be due to the suppression of inflammatory mediators leading to the resolution of lesions.

The efficacy of intralesional steroids used to successfully treat CPL has been reported in a few other cases.^8–10^ However, our response was achieved using only two injections and without concurrent treatment. Zeng et al.^8^ reported a CPL lesion on the nasal tip similar to our patient; however, they used an intralesional compound with betamethasone with concurrent treatment with intralesional interferon alpha-1b for a longer course. The report also noted telangiectasia and pigmentation on the nasal tip after 1 year of treatment. In our case, only two injections of intralesional triamcinolone acetonide were required with no concurrent treatment. In addition, our patient experienced no residual side effects after complete resolution of the lesion, and Anggraeni et al.^9^ note similar results when using intralesional triamcinolone acetonide for CPL. This may suggest that triamcinolone acetonide has better outcomes than compound betamethasone, and the discrepancies in treatment efficacy may be attributed to the differences in glucocorticoid composition but further research is needed to confirm. Conversely, Mizuno et al.^5^ reported ineffectiveness with the use of intralesional triamcinolone acetonide; however, the report does not expand on the duration, frequency, or dosing; thus, it is difficult to ascertain the reasons for ineffectiveness. Further research is needed to determine the efficacy and safety of intralesional triamcinolone acetonide use in CPL lesions.

Miguel et al.^1^ note that individualized treatment choices should be made according to etiology and localization. Previous reports of successful intralesional steroid use in treating CPL were reported in patients with lesions located on their faces.^8–10^ This may suggest that intralesional steroids should be considered especially if an individual has a CPL lesion localized to their face.

Overall, the literature on treatment for CPL is sparse, and Miguel et al.^1^ note that more cases are needed to determine effective treatment options. Our case highlights intralesional steroid injection with low-dose triamcinolone acetonide as an effective treatment. It has proven to be effective with relatively low amounts of injections and has been well tolerated by patients. Although CPL is a benign and rare disorder, it is valuable for clinicians to be cognizant of the available treatment options.
